# Analysis of Non-Ionic Surfactant Triton X-100 Using Hydrophilic Interaction Liquid Chromatography and Mass Spectrometry

**DOI:** 10.3390/molecules24071223

**Published:** 2019-03-28

**Authors:** Evelin Farsang, Violetta Gaál, Ottó Horváth, Erzsébet Bárdos, Krisztián Horváth

**Affiliations:** 1 Department of Analytical Chemistry, University of Pannonia, Egyetem utca 10, H-8200 Veszprém, Hungary; farsange@almos.uni-pannon.hu; 2 SÁGHEGY Ltd., Hutoház 040/2 HRSZ, H-9521 Kemenesszentmárton, Hungary; gaal.violetta91@gmail.com; 3 Department of General and Inorganic Chemistry, University of Pannonia, Egyetem utca 10, H-8200 Veszprém, Hungary; horvath.otto@mk.uni-pannon.hu (O.H.); bardose@almos.uni-pannon.hu (E.B.)

**Keywords:** octylphenol–polyethoxylate, non-ionic surfactant, hydrophilic interaction liquid chromatography, gradient optimization

## Abstract

It is well known that surfactants increase the solubility of hydrophobic organic compounds and cause adverse environmental effects. The removal of these compounds from the contaminated soil or ground-water is particularly difficult due to their water soluble feature. In this work, an ultra-high performance hydrophilic interaction liquid chromatographic method was developed for the separation of oligomers of Triton X-100 octylphenol-polyethoxylate non-ionic surfactant. Liquid chromatography-mass spectrometry (LC-MS) was used to identify the Triton X-100 compounds. There was a 44 mass unit difference between two adjacent peaks that is the molar mass of one ethylene oxide group (–CH${}_{2}$CH${}_{2}$O–). A quadratic retention model was applied for the estimation of retention of the examined non-ionic surfactant and the optimization of gradient elution conditions. The optimized method was suitable for the baseline separation of 28 Triton X-100 oligomers in five minutes.

## 1. Introduction

Surfactants have a wide range of applications as detergents, emulsifiers, wetting agents or dispersing agents and in households as personal-care products, cleaning products, plastics, paints, resins and pesticides [1,2]. They increase the solubility of environmentally dangerous materials (e.g., polyaromatic hydrocarbons, pesticides) and keep them in solutions. After utilization, surfactants reach the wastewater-treatment plants, where microorganisms in biological treatment phase can not degrade them, and can easily reach the natural waters [3,4,5]. Alkylphenol ethoxylates (APEOs) are one of the largest classes of non-ionic surfactants and among them, octylphenol ethoxylates (OPEOs) and nonylphenol ethoxylates (NPEOs) are the two most common used non-ionic surfactants. Triton X-100 (TX) is an octylphenol polyethoxylate, a member of the group of the non-ionic detergents.

**Figure 1:**
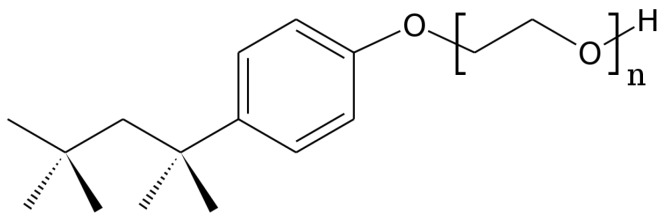
Structure of Triton X-100.

Figure 1 shows the molecular structure of the Triton X-100, where the average value of *n* is 9.5. It is a poly(ethylene glycol) terminated with a 4-(1,1,3,3-tetramethylbutyl)phenyl group at one end. The commercial alkylphenol polyethoxylates consist of homologues with different alkyl chain and oligomers with different numbers of ethylene oxide units. Its negative effect is not because of the toxicity of the compounds themselves, but rather because of the degradation potential of the decomposition products, which are able to interact with the estrogen receptor and mimic the hormones [6,7,8,9]. During the biotransformation, stepwise loss of ethylene oxide units occur in the formation of more dangerous and persistent lipophilic metabolites than the parent compound [10,11,12]. In order to understand reaction pathways, it is important to separate and identify the oligomers of Triton X-100 and the products of their degradation. In addition, determination of Triton X-100 composition is necessary for its characterization, since differences in the ethoxy chain length affect the overall viscosity, solubility, and polarity of Triton X-100 solutions.

APEOs are analyzed usually by gas chromatography (GC) coupled with flame ionization detection (FID) [13,14], electron capture detection (ECD) [15] and low-resolution mass spectrometry with electron ionization (EI) or chemical ionization (CI) techniques [16,17]. These approaches are only appropriate for APEOs with a low number of ethylene oxide groups because of volatilization issues. The GC methods for APEOs require time-consuming derivatization procedures in order to improve the volatility. Furthermore, the conversion of surfactants into volatile derivatives can be the source of errors due to the different kinetic properties of the oligomers and formation of by-products. To avoid the disadvantages, the high-performance liquid chromatography (HPLC) method has been employed with different detection systems [18]. Morales et al. [19] reviewed the extraction, clean-up, separation and quantification systems for APEOs studying the LC with fluorescence detection (LC-FD), mass spectrometry (LC-MS) and especially LC with tandem MS (LC-MS${}^{2}$). Petrovic et al. [20] studied reversed-phase liquid chromatography (RPLC) for LC-MS analysis of APEOs using silica-based C${}_{18}$ and C${}_{8}$, and alumina-based C${}_{18}$ columns which separate according to the nature of the hydrophobic moieties. Using C${}_{18}$ columns, only the alkylphenols (APs) and AP${}_{1–2}$EO could be successfully separated from other EO oligomers, which often co-eluated in one single peak. Each peak represented a single alkyl chain length and contained the whole range of polyethylene oxide chain lengths; therefore, this approach was not suitable for the analysis of the full range of APEOs. Given the complication from co-elution, Núñez et al. [21] tested normal-phase liquid chromatography (NPLC) for APEOs. NPLC was capable of analyzing the APEO oligomers according to the increasing number of EO units, while the alkyl chain length had no effect on the separation. Thus, oligomers with the same number of ethylene oxide units, but with different alkyl units, co-eluated in a single peak.

Hydrophilic interaction chromatography (HILIC) was developed by Alpert in 1990 [22]. In HILIC, polar molecules can be retained on a polar stationary phase using highly organic mobile phases containing 5–30% of aqueous buffer [23,24,25]. The higher polarity of the analyte and the less polarity of the mobile phase cause the higher retention of the polar molecules [26,27,28].

The goal of this work was to develop a gradient HILIC method for the separation of oligomers of octylphenol ethoxylate Triton X-100. The HILIC method is optimized by using the linear solvent strength model by maximizing the resolutions and minimizing the analysis time and the solvent consumption simultaneously. The retention mechanism and molecular properties of Triton X-100 oligomers are also studied in this work.

## 2. Theory

Poppe et al. [29] derived simplified equations for the calculation of retention times and peak variances in the case of linear gradients and Linear Solvent Strength (LSS) behavior. Later, Gritti et al. [30] generalized these equations for non LSS behaviors as well. The retention time of a compound can be calculated by the solution of the following differential equation:

(1) $$\frac{dz}{d\xi }=\frac{u_{0}}{k},$$

where ξ is the net time which is the time spent by a compound on the stationary phase, $\xi =t-z/u_{0}$, *z* the position of the first moment of the band (∼peak maximum) after time ξ, $u_{0}$ the linear velocity of eluent, and *t* the gross time. The integration of the above equation requires a prior knowledge of the relationship between the retention factor, *k*, and the composition of the mobile phase, φ. This relationship is almost always accounted for by an empirical quadratic equation in HILIC separations [31,32]. Accordingly:

(2) $$\ln k=\ln k_{w}+S_{1}\varphi +S_{2}\varphi^{2},$$

where $k_{w}$ is the retention factor of the compounds in the weaker eluent solvent (acetonitrile in typical HILIC separations) and $S_{1}$ and $S_{2}$ are practical measures of the sensitivity of the retention of compounds for the changes of eluent composition.

In most theoretical studies, linear gradients are considered because these have the most relevance in analytical applications and are therefore of major interest. The actual composition of the eluent at any time at the position of band of compound can be calculated as:

(3) $$\varphi =\left\{\begin{array}{cc} \varphi_{0}+\Delta \varphi \;\frac{\xi }{t_{g}}, & \mathrm{if}\;\;0\leq \xi \leq t_{g}\\ \varphi_{0}+\Delta \varphi , & \mathrm{if}\;\;\xi >t_{g}, \end{array}\right.$$

where $\varphi_{0}$ is the initial eluent composition, and Δφ the change of stronger eluent component in $t_{g}$ gradient time.

By combining Equations (2) and (3) with Equation (1) and applying the initial condition z(0)=0, the position of the compound after ξ net time can be calculated as:

z={(4a)λErfκ+S2Δφξtg−Erfκ,if  0≤ξ<tg(4b)λErfκ+S2Δφ−Erfκif  ξ=tg(4c)λErfκ+S2Δφ−Erfκ+u0kΩ(ξ−tg),if  ξ>tg,

where $k_{\Omega }$ is the retention factor of the compound at the end of gradient ($\xi \geq t_{g}$)

(5) $$k_{\Omega }=k_{w}\;\exp\left(S_{1}\left(\varphi_{0}+\Delta \varphi \right)+S_{2}{\left(\varphi_{0}+\Delta \varphi \right)}^{2}\right)$$

and

(6) $$\lambda =\frac{\sqrt{\pi }}{2}\frac{e^{\frac{S_{1}^{2}}{4S_{2}}}u_{0}}{k_{w}}\frac{t_{g}}{\sqrt{S_{2}}\;\Delta \varphi },$$

(7) $$\kappa =\frac{S_{1}+2S_{2}\varphi_{0}}{2\sqrt{S_{2}}}.$$

The critical gradient time, $t_{g,\mathrm{crit}}$, of a compound is the net retention time when it elutes together with the end of gradient exactly ($\xi =t_{g}$):

(8) $$t_{g,\mathrm{crit}}=\frac{2}{\sqrt{\pi }}\frac{k_{w}\sqrt{S_{2}}\Delta \varphi t_{0}}{e^{\frac{S_{1}^{2}}{4S_{2}}}\left(\mathrm{Erf}\left(\kappa +\sqrt{S_{2}}\Delta \varphi \right)-\mathrm{Erf}\left(\kappa \right)\right)}.$$

If $t_{g}$ is larger than $t_{g,\mathrm{crit}}$, the compound elutes before the gradient finishes. This is the usual and ideal case in gradient separations. If $t_{g}$ is smaller than $t_{g,\mathrm{crit}}$, the gradient finishes before the zone of compound leaves the column. In that case, the compound elutes with the concentration plateau of the mobile phase ($\varphi =\varphi_{0}+\Delta \varphi$).

**Figure 2:**
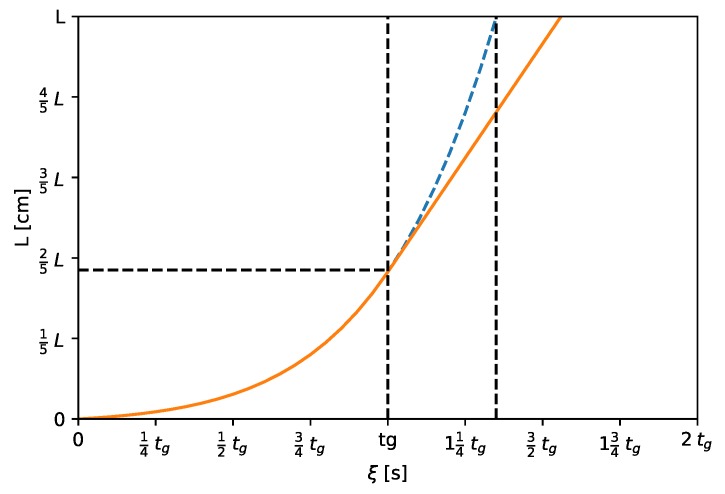
The migration of the compounds in the separation column (solid line) when the gradient time is smaller than the critical gradient time. The dashed line represents the movement of the compound if it eluted together with the end of gradient program and $t_{g}$ were equal to the critical gradient time ($t_{g,\mathrm{crit}}$).

Figure 2 demonstrates the migration of the compound in the column when its net retention time is larger than $t_{g,\mathrm{crit}}$. Three different events can be distinguished as it is shown by Equations (4a)–(4c). While ξ is less than $t_{g}$, the compound is moved forward by the gradient of the eluent. Its retention factor decreases while its migration velocity increases gradually due to the increasing ratio of stronger eluent component. In this case, the band position is defined by Equation (4a). When the end of gradient catches up the zone of compound ($\xi =t_{g}$), it is at the position defined by Equation (4b). Finally, when the gradient bypasses the zone of compound ($\xi \geq t_{g}$), the retention factor of the compound becomes constant ($k_{\Omega }$), so as its migration velocity. The band position in that case is defined by Equation (4c). In Figure 2, the solid line represents the band position throughout its migration in the separation column. The dashed line represents the movement of the compound if it were equal to the critical gradient time ($t_{g,\mathrm{crit}}$) assuming that the gradient slope, $\Delta \varphi /t_{g}$, is the same as in the case of solid line.

Considering that $\xi =t-z/u_{0}$ and $t_{0}=L/u_{0}$, the retention time of the compound (z=L) is given as:

tg={(9a)t0+κ−Erf−1Erfκ+λtgS2Δφtg,if  tg≥tg,crit,(9b)t01+kΩ+tg−λErf(κ+S2Δφ)−Erf(κ)kΩu0,if  tg<tg,crit

where ${\mathrm{Erf}}^{-1}\left(x\right)$ is the inverse error function.

In the knowledge of $k_{w}$, $S_{1}$ and $S_{2}$, the retention time of any compounds can be calculated by Equations (8) and (9a) or (9b) for a given gradient program regardless of the fact that the analyte elutes with the gradient or at the concentration plateau of the eluent.

## 3. Experimental

### 3.1. Materials

Triton X-100 octylphenol ethoxylate non-ionic detergent (C${}_{14}$H${}_{22}$O(C${}_{2}$H${}_{4}$O)${}_{9.5}$) was purchased from Alfa Aesar (Karlsruhe, Germany). Acetonitrile (Fluka) and Milli-Q Plus (Millipore, Darmstadt, Germany) ultrapure water were used as mobile phase constituents for HILIC separation.

### 3.2. UPLC System Instrumentation

HILIC separation of Triton X-100 was performed on an Agilent 1290 Infinity ultrahigh-performance liquid chromatography system (Agilent, Waldbronn, Germany) equipped with a binary high pressure pump, automatic injection system, sample manager, column thermostat and diode array detector. The volume between the pump heads and column inlet (gradient delay volume) of the UHPLC system was 0.17 mL. A Kinetex HILIC Silica (100 × 4.6 mm, 2.6 μm) analytical column (Phenomenex, Torrance, CA, United States) packed with a core–shell silica stationary phase was utilized.

The flow rate of the eluents was 1 mL/min ($t_{0}$ = 0.99 min), the column temperature was 20 ${}^{\circ }$C and the injection volume was 2 μL. The chromatographic elution was conducted with binary mobile phase consisting of an organic part (A): acetonitrile (ACN) and an aqueous part (B): 2% ACN in 98% water. During elutions, a linear gradients were run from 100% to 50% A (Δφ=0.49). The gradient times were 3.0, 3.5, 4.0, 4.5, 5.0, 5.5, and 6.0 min. The detection wavelengths were 200, 223 and 275 nm. Absorbance spectra of Triton X-100 have maximums at these wavelengths. The injection of the sample was delayed by 0.17 min that corresponds to the gradient delay time of the system. By applying this injection delay, the sample arrives to the column when the gradient starts. As sample preparation, 1000 μL of acetonitrile was added to 10 μL of Triton X-100.

The chromatograms were recorded by Agilent Chemstation for LC 3D system version B.04.03 and were evaluated by Mathematica 10.0 (Wolfram Research Inc., Champaign, IL, United States), Peakfit (Systat Software Inc., Saint Jose, CA, United States, version 4.12), Gnuplot (version 4.6, http://www.gnuplot.info) and algorithms written in Python programming language (v. 3.6, Anaconda Python Distribution, https://anaconda.com), using the NumPy and SciPy packages.

### 3.3. LC-MS System Instrumentation

LC-MS separations were performed on an Aquity UPLC H-Class System (Waters, Milford, MA, United States) consists of a Quaternary Solvent Manager (QSM), a Sample Manager-Flow Through Needle (SM-FTN), a column heater, a Photodiode Array Detector (PDA) and a single quadrupole mass detector (QDA) with electrospray ionization. A Kinetex HILIC Silica analytical column (100 × 4.6 mm, 2.6 μm), packed with a core–shell silica stationary phase was utilized. The column temperature was set to 20 ${}^{\circ }$C. Eluent A was 100% ACN, eluent B was 2% ACN in water. Both of the eluents contained 100 μL/L formic acid in order to improve peak shapes during the separation. The flow rate was 1 mL/min. The injection volume was 2 μL. The ionization was done in positive mode with +20 V cone voltage. Initial gradient condition was set to A:B = 100:0% before embracing a linear gradient increase to 50% B. The gradient time was set to 4 min. UV detection was done on the wavelength of 200 nm.

### 3.4. Computation

All of the computational work was performed by using algorithms written in-house in Python programming language with NumPy and SciPy scientific libraries. For the determination of $k_{w}$, $S_{1}$ and $S_{2}$ values, Equation (9) was fitted on a retention database that consisted of retention time against gradient time data pairs. curve_fit function provided by the SciPy library was used for the curve fitting. A Levenberg–Marquardt algorithm was used for the minimization. Usually, it is the most efficient method for small unconstrained problems. The figures were generated by the Matplotlib Python library.

## 4. Results and Discussion

### 4.1. Peak Identification Using the LC-MS Method

HILIC LC-MS was used to identify the Triton X-100 compounds applying gradient separation (see Section 3). In Table 1, the *m*/*z* values of the first 14 TX components can be seen. The *m*/*z* ratio of isotope peaks confirmed that the molecules had one charge. Taking the *m*/*z* ratio, isotopic peak and molecular structure into account, we can conclude that a sodium ion binds to the hydrophilic polyethylene oxide group ([M + Na]${}^{+}$). This phenomena is common in LC-MS separations because glass containers may leach sodium contamination at very low concentrations. It can be seen that there is a 44 mass unit difference between two adjacent peaks that is exactly the molar mass of one ethylene oxide (EO) group (–CH${}_{2}$CH${}_{2}$O–).

### 4.2. Retention Behavior of Triton X-100 Components

**Figure 3:**
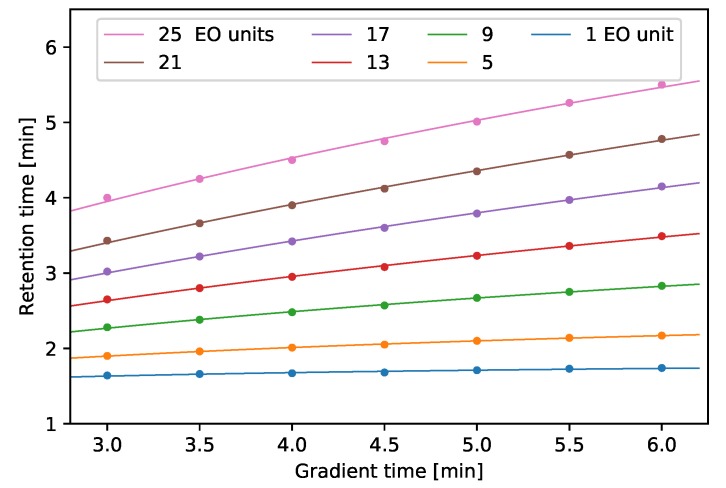
Retention times of Triton X-100 compounds at different gradient times. The number of ethylene oxide units are presented in the legend. Dots and lines represent measured and calculated data, respectively.

Preliminary isocratic experiments (volume ratios of the acetonitrile were 50%, 55%, 60%, 64%, 68%, 72%, 76%, 80%, 84%, 88%, 90%, 92%, 94% and 96%, results are not shown here) revealed that isocratic HILIC separations were not suitable for the resolution of all Triton X-100 oligomers in a reasonable analysis time. Even the weakest eluent (96% acetonitrile) was not able to resolve the smallest TX compounds, while the retention time of the 17th peak was slightly less than 20 min. Triton X-100 is a good example for the general elution problem [32], which is one of the main reasons for using gradient elutions in HPLC. As it was shown in the Theory section, optimization of gradient separation requires the knowledge of $k_{w}$, $S_{1}$ and $S_{2}$ parameters of all the TX compounds. In order to determine these parameters, retention times of TX compounds were recorded at different gradient runs. The gradient times, $t_{g}$s, were systematically changed between 3.0 and 6.0 min (for details, see Section 3). The 50–50% water:ACN isocratic measurements data were used as the retention times belonging to $t_{g}=0$ gradient time. As it can be seen in Figure 3, the retention times increased as the time of gradient increased.

Equation (9) was fitted on the measured retention times in order to determine values of $k_{w}$, $S_{1}$ and $S_{2}$ parameters. Similarly to the methylene selectivity of reversed phase stationary phases, it was assumed that the HILIC phase had a constant ethylene oxide selectivity, $\alpha_{\mathrm{EO}}$. Accordingly, $k_{w}$ values of successive members of the homologous series of TX compounds differ by a factor of $\alpha_{\mathrm{EO}}$:

(10) $$k_{w,n}=\alpha_{\mathrm{EO}}k_{w,n-1}=\alpha_{\mathrm{EO}}^{n-1}k_{w,1},$$

where *n* represents the number of EO units in a Triton X-100 compound.

During the fitting procedure, Equation (9) was fitted on all the data series simultaneously. The goodness of fitting can be confirmed by close examination of Figure 3. The calculated and measured retention times are indicated by continuous lines and dots, respectively. It can be seen that the calculated lines fit well on the measured data points. The slope, intercept, and $r^{2}$ of the linear regression line fitted on the calculated and measured data are 0.9998, 0.00037, and 0.99994, respectively. The average absolute difference between the calculated and measured retention times were 0.0053 min with 4.95 × 10${}^{-5}$ min minimal and 0.037 min maximal deviations. Accordingly, it can be concluded that the quadratic retention model defined in Equation (2) could be used in this HILIC system for modeling gradient separations and the determined $\alpha_{\mathrm{EO}}$, $k_{w,1}$, $S_{1}$ and $S_{2}$ values describe the retention behavior of the studied molecules correctly.

**Figure 4:**
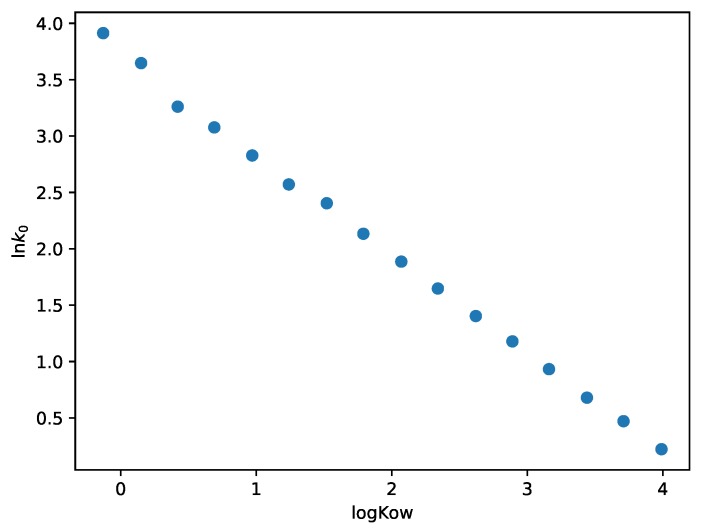
Logarithm of $k_{0}$ as a function of octanol/water partition coefficient.

The hydrophobicity of a compound can be characterized by the octanol/water partition coefficient ($\log K_{ow}$), which is the ratio of the analyte concentration in octanol and in water. In case of a shorter poly(ethylene glycol) chain, higher hydrophobicity can be observed. Figure 4 shows the values of $\ln k_{0}$ as a function of octanol/water partition coefficient [5]. It can be seen that the relationship between the two parameters is linear. The increasing value of $\log K_{ow}$ causes a monotonic decrease in the value of $\ln k_{0}$.

**Figure 5:**
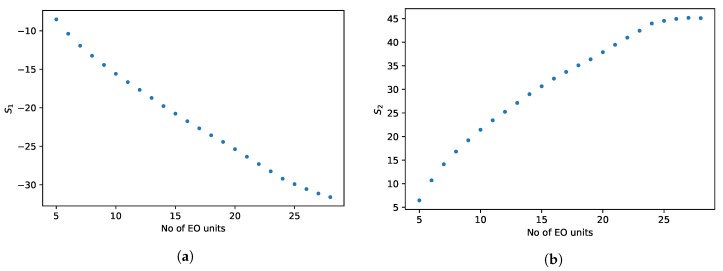
Retention sensitivities $S_{1}$ (**a**) and $S_{2}$ (**b**) of the Triton X-100 compounds as the function of the number of ethylene oxide units.

In Figure 5a the $S_{1}$ and in Figure 5b the $S_{2}$ parameters of TX compounds can be seen as a function of the number of ethylene oxide units. As Figure 5a shows, values of $S_{1}$ parameters are negative, which means that most probably there is a competition between the analyte and the water molecules for the bindings sites of the stationary phase. In addition, $S_{1}$ values decrease monotonically with the number of EO units, which means that the sensitivity of the compounds toward the change of eluent composition increases as the sizes of molecules increase. A similar phenomenon can be observed e.g., in ion chromatography. While in ion chromatography the sensitivity depends on the charge of the analyte, in HILIC, the increasing molecular size enhances the sensitivity [33].

Values of $S_{2}$ parameters (Figure 5b) are positive. Accordingly, water can enhance the adsorption of TX compounds as well. Similarly to the competitive parameter, $S_{1}$, increasing values of $S_{2}$s show that the collaborating effect of water is higher for larger molecules.

**Figure 6:**
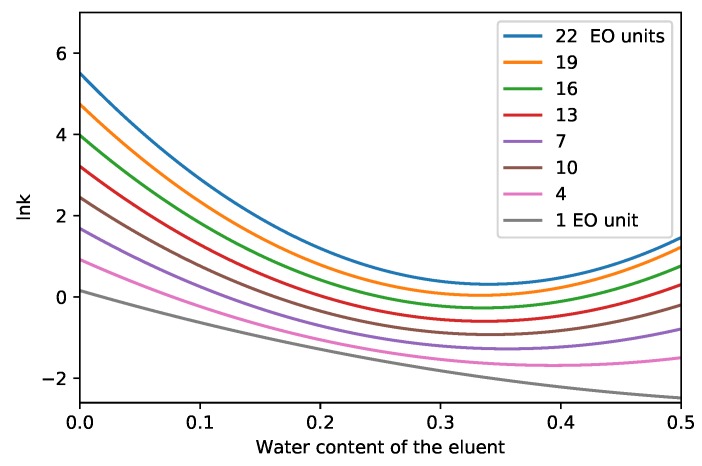
Logarithm of retention factor at different changes of the eluent conditions. The number of ethylene oxide units are represented in the legend.

Figure 6 shows the logarithm of retention factors as a function of eluent composition. By changing the water content of the eluent from 0% to 50%, U-shaped lnk vs. φ curves can be observed. Increasing the water content up to 40% causes the decrease of retention factors. It can be explained by the strongest eluting property of water, which is a common phenomena in HILIC separations [22,26]. However, further addition of water increases the retention factor. This trend has not been explained yet on a strong theoretical basis, but was observed by several research groups (e.g., [34,35,36]). It can be seen that the U-shape is more significant for larger molecules than for small ones. Figure 6 serves with the important conclusion that the water content of eluent should not exceed 40% during the gradient separation of Triton X-100 compounds. A further increase would increase retention times and would make the eluting peaks wider.

**Figure 7:**
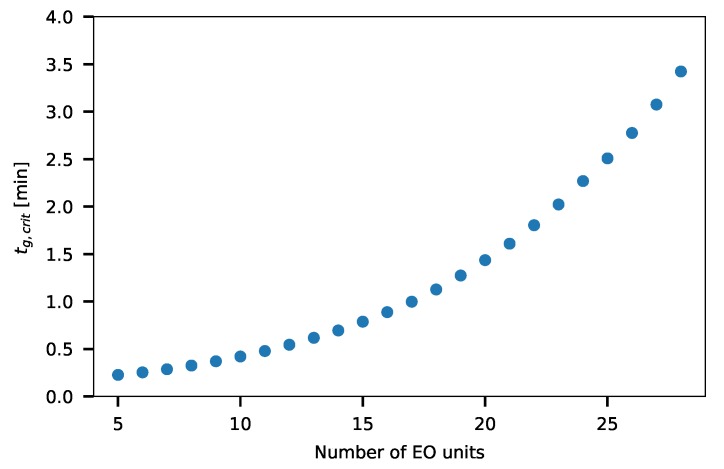
The critical gradient time of the Triton X-100 compounds with a different number of ethylene oxide units (Δφ = 0.42 and $\varphi_{0}$ = 0).

Figure 7 shows the critical gradient times, $t_{g,\mathrm{crit}}$s, of the Triton X-100 compounds in case of Δφ = 0.42 and $\varphi_{0}$ = 0 values. It can be seen that $t_{g,crit}$ is less than four minutes for every examined compound. It means that all compounds elute before the gradient program finishes if the gradient time is less than four minutes.

### 4.3. Optimization of Gradient Separations

**Figure 8:**
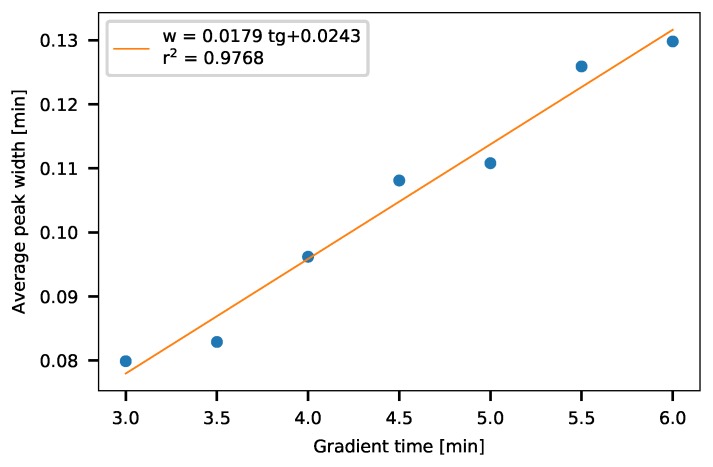
Average peak width depending on the gradient times.

Based on the gradient measurements, peak widths of TX compounds were determined by peak fitting using PeakFit software. The determined peak widths were averaged for each gradient time. Figure 8 shows that the average peak widths change linearly with the gradient time. By fitting a linear line on the measured peak widths, these can be calculated as:

(11) $$w=0.0179\;t_{g}+0.0243.$$

In the knowledge of retention times and peak widths, resolutions, $R_{s}$, can be calculated:

(12) $$R_{s}=2\frac{|t_{B}-t_{A}|}{w_{A}+w_{B}}≃\frac{|t_{B}-t_{A}|}{w},$$

where $t_{A}$ and $t_{B}$ can be calculated by Equation (9), and *w* by Equation (11).

**Figure 9:**
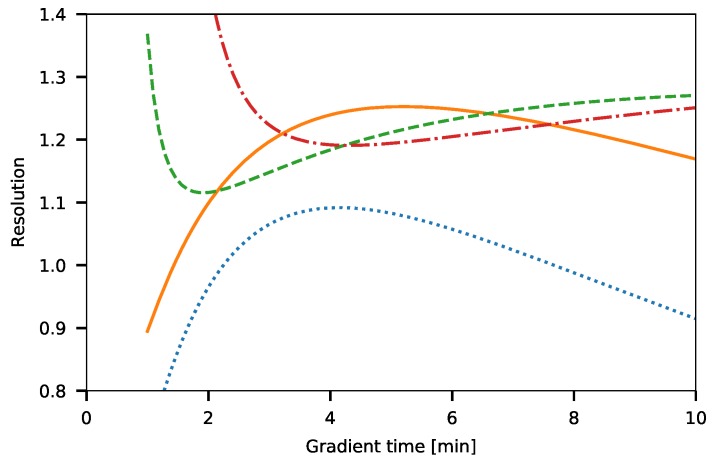
A resolution of different Triton X-100 oligomer pairs. The number of ethylene oxide units: 5/6 (dotted line), 8/9 (solid line), 15/16 (dashed line), and 21/22 (dashed-dotted line).

In Figure 9, resolution of adjacent peaks (5–6, 8–9, 15–16, 21–22 number of EO units) are shown. It can be seen that the resolutions have a maximum value for compounds below 10 EO units and a minimum value above them. It can be stated that using a 4-min long gradient program appropriate resolution ($R_{s}\geq 1.1$) can be achieved.

**Figure 10:**
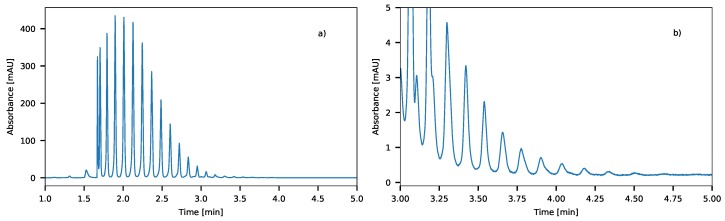
Full (**a**) and zoomed (**b**) chromatograms of Triton X-100 compounds applying a 4-min long gradient (λ = 200 nm, Δφ = 0.42 and $\varphi_{0}$ = 0).

Based on Figure 9, it can be concluded that the optimal gradient time for the separation of Triton X-100 compounds is four minutes. Figure 10 shows a chromatogram of the optimal separation. A suitable separation of TX compounds were achieved by the optimized gradient. On the chromatogram, 28 different oligomers can be detected.

## 5. Conclusions

Surfactants mobilize toxic compounds and increase their harmful effect on human health by biological accumulation and concentration through the food chain system. Determination and control of these compounds require the development of high performance analytical methods. In this work, an ultra-high performance hydrophilic interaction liquid chromatographic method was developed for the separation of Triton X-100 oligomers. Mass spectrometry was successfully applied to identify the compounds. A quadratic empirical retention model was applied for the estimation of retention behavior of solute molecules. Gradient retention equations were developed taking into consideration the scenario when the gradient program finishes before the compounds elute. The developed gradient model allowed the optimization of elution conditions. With the optimized method, 28 Triton X-100 oligomers were separated in less than five minutes. 

## Figures and Tables

**Table 1:** First 14 identified Triton X-100 compounds.

*m*/*z*	Formula	No. EO Units
361.27	C${}_{20}$H${}_{34}$O${}_{4}$Na${}^{+}$	3
405.34	C${}_{22}$H${}_{38}$O${}_{5}$Na${}^{+}$	4
449.34	C${}_{24}$H${}_{42}$O${}_{6}$Na${}^{+}$	5
493.38	C${}_{26}$H${}_{46}$O${}_{7}$Na${}^{+}$	6
537.44	C${}_{28}$H${}_{50}$O${}_{8}$Na${}^{+}$	7
581.49	C${}_{30}$H${}_{54}$O${}_{9}$Na${}^{+}$	8
625.53	C${}_{32}$H${}_{58}$O${}_{10}$Na${}^{+}$	9
669.58	C${}_{34}$H${}_{62}$O${}_{11}$Na${}^{+}$	10
713.59	C${}_{36}$H${}_{66}$O${}_{12}$Na${}^{+}$	11
757.65	C${}_{38}$H${}_{70}$O${}_{13}$Na${}^{+}$	12
801.69	C${}_{40}$H${}_{74}$O${}_{14}$Na${}^{+}$	13
845.72	C${}_{42}$H${}_{78}$O${}_{15}$Na${}^{+}$	14
889.76	C${}_{44}$H${}_{82}$O${}_{16}$Na${}^{+}$	15
933.76	C${}_{46}$H${}_{86}$O${}_{17}$Na${}^{+}$	16

## Abbreviations

The following abbreviations are used in this manuscript:

TX	Triton X-100
HILIC	Hydrophilic Interaction Liquid Chromatography
LC MS	Liquid Chromatography Mass Spectometry
APEO	Alkylphenol Ethoxylate
OPEO	Octylphenol Ethoxylate
NPEO	Nonylphenol Ethoxylate
GC	Gas Chromatography
FID	Flame Ionization Detection
ECD	Electron Capture Detection
EI	Electron Ionization
CI	Chemical Oonization
HPLC	High Pressure Liquid Chromatography
FD	Fluorescence Detection
NPLC	Normal Phase Liquid Chromatography
LSS	Linear Solvent Strength
